# Immune Checkpoint Inhibitors in the Management of Brain Metastases from Non-Small Cell Lung Cancer: A Comprehensive Review of Current Trials, Guidelines and Future Directions

**DOI:** 10.3390/cancers16193388

**Published:** 2024-10-03

**Authors:** Tulika Ranjan, Vivek Podder, Kim Margolin, Vamsidhar Velcheti, Arun Maharaj, Manmeet Singh Ahluwalia

**Affiliations:** 1 Miami Cancer Institute, Baptist Health South Florida, Miami, FL 33186, USA; dr.ranjan2017@gmail.com (T.R.); drvivekpodder@gmail.com (V.P.); arun.maharaj@baptisthealth.net (A.M.); 2 Saint John’s Cancer Institute, Santa Monica, CA 90404, USA; kim.margolin@providence.org; 3 NYU Langone’s Perlmutter Cancer Center, New York, NY 10016, USA; vamsidhar.velcheti@nyulangone.org

**Keywords:** brain metastasis, non-small cell lung cancer, immune checkpoint inhibitors, combination therapy, radiotherapy

## Abstract

**Simple Summary:**

Immune checkpoint inhibitors (ICIs) have emerged as a promising treatment for brain metastases (BM) in non-small cell lung cancer (NSCLC), offering improved survival outcomes. Despite their potential, challenges remain due to the complex nature of BM and the need for combination therapies, including radiotherapy and anti-VEGF agents. This review discusses the current role of ICIs, the importance of optimizing treatment sequences, and the management of side effects. A personalized, multidisciplinary approach is essential for maximizing the benefits of ICIs in NSCLC patients with BM.

**Abstract:**

Background: Brain metastases (BM) are a common, severe complication in patients with non-small cell lung cancer (NSCLC) and are difficult to treat due to their complex tumor biology and the intricate microenvironment of the brain. Objectives: This review examines the current role of immune checkpoint inhibitors (ICIs) in treating NSCLC with BM, focusing on the latest clinical trials, emerging strategies, current guidelines, and future directions. We highlight the efficacy of ICIs as monotherapy and in combination with other treatments such as radiotherapy, stereotactic radiosurgery, chemotherapy, and anti-VEGF agents. Results: While no single treatment sequence is universally accepted, combining ICIs with traditional therapies forms the core of the current treatment protocols. ICIs targeting the PD-1/PD-L1 pathway have significantly advanced NSCLC treatment, demonstrated by improved overall and progression-free survival in various settings. However, optimizing these benefits requires careful consideration of potential side effects, including cognitive decline and radiation necrosis, and the impact of steroid use on ICI efficacy. Conclusion: The review underscores the necessity for a personalized, integrated multidisciplinary treatment approach. Future research should focus on refining combination therapies and understanding the optimal sequence and timing of treatment.

## 1. Introduction

Brain metastases (BM) diagnosed in patients with non-small cell lung carcinoma (NSCLC) significantly impacts survival outcomes and prognosis. NSCLC accounts for 40–50% of all BMs worldwide, emphasizing the critical need for prompt diagnosis and effective treatment strategies. Approximately 55–60% of NSCLC patients are diagnosed with metastatic disease, and around 20% present with BM at initial diagnosis [1]. In 35% of stage IV NSCLC cases, BMs are the sole site of disease spread [1], and the progression of NSCLC increases the likelihood of developing BM, affecting 25% to 50% of patients [2,3,4,5]. Patients with small cell lung cancer (SCLC) are twice as likely to develop BM compared to those with NSCLC. At diagnosis, about 20% of SCLC patients have BM, and an increased prevalence of up to 80% showing central nervous system (CNS) involvement within two years. The management of BM in NSCLC is challenging due to the tumor’s biology, the unique properties of the brain microenvironment, and the difficulty in delivering effective treatments across the BBB [5].

Comprehensive molecular profiling is vital for choosing the optimal initial treatment strategy for advanced NSCLC. Agents targeting the actionable mutations can improve overall survival (OS). Without targetable mutations, treatments involve immune checkpoint inhibitors (ICIs) alone or combined with chemotherapy to target key proteins like Programmed Death 1 (PD-1), PD-ligand 1 (PD-L1), and cytotoxic T-lymphocyte-associated protein 4 (CTLA-4), which has advanced the management of NSCLC with BM. ICIs such as pembrolizumab, nivolumab, atezolizumab, and ipilimumab block these immune checkpoints, allowing T cells to recognize and attack tumor cells effectively. This mechanism of action restores anti-tumor immunity by reversing immune escape and is particularly effective in countering the immunosuppressed tumor microenvironment of the brain. These protein-specific therapies have shown efficacy in activating the brain’s immune response, a site traditionally considered challenging due to its immunologically unique milieu [1,5]. The effectiveness of ICIs in NSCLC is well documented, with several clinical trials demonstrating significant improvements in overall survival (OS) and progression-free survival (PFS) rates, with superior OS in patients with high PD-L1 expression (≥50%) [5].

This article reviews the use of ICIs in NSCLC with BM and explores the recent clinical trials, National Comprehensive Cancer Network (NCCN) guidelines, recent developments, and future directions. We review the effectiveness of both single-agent and dual-agent ICIs, their integration with radiotherapy, stereotactic radiosurgery (SRS) and chemotherapy, and the factors affecting ICI efficacy, including steroid use and vascular endothelial growth factor (VEGF) inhibitors. Lastly, we offer insights into the evolving ICI-based treatment strategies and the importance of clinical trials with a multimodal approach, contributing to improved management approaches for BM in NSCLC in the immunotherapy era.

## 2. Integrated Therapeutic Strategies for Brain Metastases in NSCLC

Currently, there are no universally accepted treatment recommendations for BM, resulting in inconsistent treatment approaches. Surgical resection, radiotherapy, and systemic therapies are the mainstays in managing BM. Surgical resection in patients with BM is typically considered for managing mass effects or symptoms to treat patients with newly diagnosed or stable systemic disease, or for reasonable systemic treatment options with limited intracranial disease [6].

Radiotherapy treatment has become more conformal, allowing ablative doses to be delivered precisely to the metastatic areas. This approach provides greater local control and reduces neurological toxicity. Options for those with multiple BMs or a solitary BM with or without resection include SRS/stereotactic radiotherapy (SRT) and whole brain radiotherapy (WBRT) with or without hippocampal avoidance and with or without memantine [6,7,8]. Radiation induces an ischemia–hypoxia cascade, increasing the glutamate levels and activating the N-methyl-D-aspartate (NMDA) receptors, leading to excitotoxicity and cell death. As an NMDA receptor antagonist [6,7,8], memantine blocks excessive glutamate activity, protecting against excitotoxicity and preserving cognitive function during WBRT.

After localized treatment like surgery and/or radiotherapy, systemic therapies such as chemotherapy, targeted therapy, immunotherapy, or combined modalities should be considered, especially in patients with targetable genetic alterations, because systemic therapies can address micrometastases that might not be detectable with imaging or controlled by local therapies alone. These micrometastases pose a risk of subsequent metastatic spread, and early systemic intervention could play a critical role in preventing further dissemination of the disease. The incorporation of systemic therapies following localized treatments aims not only to manage the existing disease but also to interrupt the metastatic process by targeting circulating tumor cells or residual tumor cells at a microscopic level.

## 3. The Role of Immunotherapy as a Game-Changer in NSCLC Brain Metastasis

**Figure 1:**
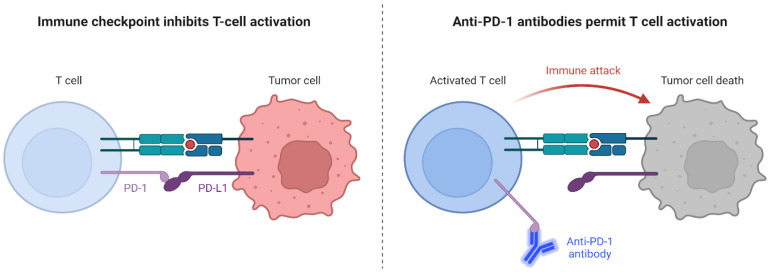
Mechanisms of Action of Immune Checkpoint Inhibitors on Tumor Cells. Created with BioRender.com.

ICIs have emerged as a frontline treatment strategy of NSCLC. ICIs activate the immune response within the immunosuppressive tumor microenvironment of BM [9] by targeting specific proteins on the surface of cancer cells (PD-L1) or immune cells (PD-1), maintaining a normal immune response (Figure 1). By inhibiting these checkpoints, ICIs assist the immune system in effectively recognizing and eliminating cancer cells [10].

To date, approved ICIs for the frontline treatment of metastatic NSCLC include PD-1 inhibitors (pembrolizumab, nivolumab, and cemiplimab), PD-L1 inhibitors (atezolizumab), and CTLA-4 inhibitors (ipilimumab) [6]. Several clinical trials and retrospective studies have evaluated the efficacy of ICIs in treating NSCLC with BM, as summarized in Table 1. Randomized trials have shown that high PD-L1 expression (≥50%) predicts response to pembrolizumab, atezolizumab, or cemiplimab in NSCLC with BM [11]. These results are clinically relevant in that approximately 30% of advanced NSCLCs exhibit this level of PD-L1 expression [11]. ICI treatment options include ICI as monotherapy or dual therapy and chemoimmunotherapy, leveraging the predictive value of PD-L1 levels for effective patient management.

The effective use of either PD-1 or PD-L1 inhibitors, alone or combined with chemotherapy or anti-CTLA-4 agents, has been observed in NSCLC and SCLC patients with BM [6,12,13,14]. The NCCN 2023 guidelines recommend treatment for stage IV NSCLCs based on PD-L1 expression levels, histology, and the patient’s performance status (PS) [6]. Single-agent pembrolizumab is recommended for stage IV non-squamous cell carcinoma (non-SCC) and squamous cell carcinoma (SCC) with high PD-L1 expression (Tumor Proportion Score (TPS) ≥ 50%) and a PS of 0 to 1. In cases of stage IV non-SCC with negative (<1%), low (1–49%), or unknown PD-L1 levels, a combination of pembrolizumab, carboplatin, and pemetrexed (for non-SCC) or carboplatin with paclitaxel or nab-paclitaxel (for SCC) is advised [6]. These guidelines emphasize the importance of PD-L1 expression in determining the most effective frontline approach for each patient, especially in non-SCC NSCLC. However, it is important to note the limitations of PD-L1 as a biomarker in non-SCC NSCLC. PD-L1 expression can sometimes be high in patients with oncogene driver-positive alterations, such as epidermal growth factor receptor (EGFR) mutations and other driver alterations where the responses to ICIs are typically poor [6]. These patients should instead be treated with targeted therapies. In non-SCC NSCLC, molecularly targeted therapies are important due to their ability to leverage specific genetic alterations typical of this subtype. This targeted approach is crucial as these therapies can provide significant benefits over traditional chemotherapy, particularly for patients whose tumors have specific, actionable mutations.

**Table 1:** Pivotal Clinical Trials of ICI Monotherapy and Dual Therapy in NSCLC Brain Metastases.

Trial Name	Study Design	Patient Population (with BM)	Intervention Arm	Control Arm	PD-L1	iORR (num. %)	mPFS	mOS
Studies with ICI Monotherapy								
OAK Trial [15]	Phase III	61 (A) 62 (B) (treated asymptomatic BM)	Atezolizumab (A)	Docetaxl (B)	NR	13.7 (A), 11.8 (B)	NR	16 (A), 11.9 (B)
FIR Trial [16]	Phase II (single arm)	13 (treated asymptomatic BM)	Atezolizumab	None	>5%	23	2.5	6.8
JAVELIN Lung 200 Trial [17,18]	Phase III	46 (A), 33 (B) (treated asymptomatic BM)	Avelumab (A)	Docetaxel (B)	PD-L1 ≥ 1%	18.9 (A), 10.6 (B) [PD-L1+]	NR	NR
Goldberg SB, et al. [19,20]	Phase II	37 (asymptomatic BM)	Pembrolizumab	None	Cohort 1: PD-L1 ≥ 1%, Cohort 2: PD-L1 < 1%	18.9	1.9	9.9
Wakuda K, et al. [21]	Retrospective	23	Pembrolizumab	None	TPS ≥ 50%	54 (treated BM), 60 (untreated BM)	6.5 (treated BM), 5.3 (untreated BM)	21.6 (entire BM population)
EMPOWER-Lung 1 [22,23]	Phase III	68 (stable, treated BM)	Cemiplimab (A)	Chemotherapy (investigator’s choice) (B)	≥50%	41.2 (A), 8.8 (B)	10.4 (A), 5.3 (B)	18.7 (A), 11.7 (B)
Crino L, et al. [24]	EAP, outside controlled clinical trial	409 (asymptomatic BM)	Nivolumab	None	NR	19	3	8.6
Assié JB, et al. [25]	Retrospective	1800	Nivolumab	None	NR	NR	NR	9.9
Devieuvre D et al. [26]	Retrospective	477	Nivolumab	None	NR	NR	NR	9.7 (with BM), 11.9 (without BM)
Grossi F, et al. [27]	Retrospective	409	Nivolumab	None	NR	17	3	8.6
**Studies with ICI Dual Therapy**								
CheckMate 227 [28]	Phase III	69 (A), 66 (B) (asymptomatic untreated BM)	Nivolumab and ipilimumab (A)	Chemotherapy (B)	>1% and TMB > 10	33 (A), 26 (B)	5.4 (A), 5.8 (B)	18.8 (A), 13.7 (B)
Checkmate 817 [29]	Phase IIIb/IV	59 (untreated BM)	Nivolumab with weight-based ipilimumab	None	Any	37	4.2	NR

A and B: Denote different arms within the study (e.g., treatment or control arms as defined in the study protocol; NR: Not Reported; PD-L1+: PD-L1+: Defined as PD-L1 expression ≥ 1% of tumor cells.

## 4. Single-Agent Immune Checkpoint Inhibitors in NSCLC with Brain Metastases

Single-agent ICIs that target the PD-1/PD-L1 pathways, such as pembrolizumab, nivolumab, cemiplimab, and atezolizumab, have significantly improved OS and PFS in advanced NSCLC patients with BM [30,31]. Survival is more prominent when combined with other agents and modalities, such as WBRT or SRT. The following sections provide a summary of the trials that include patients with previously treated, stable, and steroid-free BM alone, as well as the trials that include subjects with active but asymptomatic and steroid-free and/or active but symptomatic and/or steroid-dependent BM (Table 1). Other malignancies were used as examples or comparisons relative to NSCLC where appropriate. The following trials refer to a mixture of both subtypes when NSCLC is mentioned without specifying SCC or non-SCC.

### 4.1. Pembrolizumab: Pivotal Clinical Trials and Survival Benefits

Pembrolizumab is a humanized anti-PD-1 antibody approved for advanced NSCLC and has demonstrated marked efficacy in the KEYNOTE clinical trials. In these studies, patients treated with pembrolizumab had an improved OS compared to those treated with chemotherapy, especially in tumors with high PD-L1 expression [11,32,33,34]. In a pooled analysis of 3170 NSCLC patients (9.2% with treated and stable BM), those treated with pembrolizumab had a greater OS than those receiving chemotherapy, regardless of whether PD-L1 expression (reported as TPS) was ≥50% or, less stringently, ≥1% [35]. An analysis by Goldberg et al., including 37 NSCLC patients with previous systemic treatment who relapsed with BM (measuring 5–20 mm) and PD-L1 ≥ 1%, showed a 29.7% CNS overall response rate (ORR) with pembrolizumab [19]. In this study, 16 patients experienced progressive disease (PD), with 6 unevaluable due to systemic progression. The median to CNS response was 1.8 months (interquartile range, IQR: 1.7 to 2.4 months) as evaluated by the modified Response Evaluation Criteria in Solid Tumors (mRECIST), with a response duration of 5.7 months. Discordance in the cranial–extracranial responses was noted in 6 out of 27 evaluable patients, where the brain was the progression site in 3 patients who otherwise responded systemically and vice versa. Further, all discordant cases survived over 6 months, including 2 patients with a partial systemic response but progressive CNS disease exceeding 2 years. The study reports a median PFS of 1.9 months and OS of 9.9 months, with a 2-year OS rate of 34%. Results from this study demonstrate the differential efficacy of pembrolizumab in CNS versus systemic disease. Findings from this pivotal study emphasize the need for integrating treatment strategies aimed at optimizing outcomes across both CNS and systemic disease.

### 4.2. Nivolumab: Pivotal Clinical Trials and Survival Benefits

Nivolumab is a fully humanized immunoglobulin G4 (IgG4) anti-PD-1 antibody approved for treating advanced or metastatic NSCLCs. The effectiveness of this treatment has been demonstrated in two important clinical trials. In these trials, the proportion of BM patient involvement (treated and stable) was reported in 6.25% (*n* = 17) and 11.68% (*n* = 68), respectively [36,37]. In the phase III CheckMate-057 trial involving 292 patients with non-SCC NSCLC, nivolumab showed superior efficacy over docetaxel, with a median OS of 12.2 months and an ORR of 19% [36]. Although nivolumab had a shorter median PFS (2.3 months vs. 4.2 months), its one-year PFS rate (19% vs. 8%) was more favorable than docetaxel. Both groups received prior platinum-based chemotherapy, EGFR tyrosine kinase inhibitors, and anaplastic lymphoma kinase (ALK) inhibitors. Conversely, the CheckMate-017 phase III trial included 272 patients with advanced squamous NSCLC and those with treated stable BM. Nonetheless, results from this study showed that nivolumab improved median OS to 9.2 months versus 6.0, a one-year OS rate of 42% compared to 24% and an ORR of 20%, alongside a longer median PFS [37]. Both groups had received platinum-based chemotherapy, gemcitabine, paclitaxel, etoposide, vinorelbine, pemetrexed, fluorouracil, bevacizumab, and cetuximab. In both the CheckMate trials, nivolumab demonstrated its efficacy by OS and PFS in patients with non-squamous and squamous NSCLC, respectively, highlighting its broad applicability across histological subtypes of NSCLC.

NSCLC patients, 409 of which had asymptomatic BM who were either neurologically stable while off corticosteroids, or on a stable or decreasing dose of ≤10 mg/day prednisone [24]. Disease status was assessed every 8–12 weeks using a total body computed tomography (CT) scan (brain, chest, and abdomen) or chest and abdomen CT and brain magnetic resonance imaging. Nivolumab had an ORR of 17%, median PFS of 3 months, and median OS of 8.6 months in the CNS group. In the CNS subgroup, the one-year OS rate was 43%, closely aligned with the 48% observed in the entire cohort. The disease control rate (DCR)—defined as the combined rates of CR, PR, and SD—was 40% in the CNS subgroup compared to 44% in the overall group, indicating that Nivolumab’s efficacy in patients with BM was comparable to that seen in the broader cohort of patients without BM. Nivolumab has thus emerged as a key therapeutic agent for NSCLC, demonstrating efficacy in patients without BM and those with asymptomatic BM. Further studies exploring its use in combination with other therapies and in diverse patient populations will help refine its role and potentially expand its therapeutic reach.

### 4.3. Cemiplimab: Pivotal Clinical Trials and Survival Benefits

Cemiplimab is a humanized anti-PD-1 monoclonal antibody approved for first-line treatment of NSCLC with high PD-L1 (≥50%) and no EGFR, ALK, or ROS1 aberrations, used alone or with platinum-based chemotherapy in advanced or metastatic cases in patients unsuitable for surgery or definitive chemoradiation [38]. In the EMPOWER-Lung 1 trial, cemiplimab was tested in 68 patients as a frontline treatment of advanced NSCLC and treated stable BM [38]. The ORR of cemiplimab were found to be positive in patients whose PD-L1 was ≥50%. Cemiplimab showed superior PFS and OS benefits over chemotherapy (platinum-based doublet chemotherapy, including pemetrexed, cisplatin, gemcitabine, paclitaxel, and carboplatin) for the entire PD-L1 ≥ 50% cohort and specifically for those with BM. In the BM subgroup, the hazard ratios (HRs) for median OS and median PFS were notably low at 0.17 (95% confidence interval, CI: 0.04–0.76) and 0.45 (95% CI: 0.22–0.92), respectively. Further, post hoc analysis revealed an intracranial (IC) PFS of 72% and IC disease progression of 5.9% in the cemiplimab group compared to chemotherapy (11.8%). Results from this analysis suggest a potential benefit from cemiplimab treatment use, but more evidence is warranted to validate this observation.

### 4.4. Atezolizumab: Pivotal Clinical Trials and Survival Benefits

Atezolizumab is an anti-PD-L1 humanized monoclonal antibody approved for treating metastatic NSCLC. The OAK trial was a phase III study evaluating the use of atezolizumab, involving 1225 previously treated NSCLC patients, of whom 123 had treated asymptomatic BM. Atezolizumab demonstrated a non-significant trend towards improved OS (16 vs. 11.9 months) compared to docetaxel, with two-year OS rates of 26.6% vs. 19.3% [16]. Further, atezolizumab significantly delayed the radiographic identification of new onset of symptomatic brain lesions in patients with previously treated, asymptomatic BM, with the median time not reached in the atezolizumab group compared to 9.5 months in the docetaxel group [16]. In addition, the phase II FIR trial was conducted using atezolizumab in 138 NSCLC patients with >5% PD-L1 expression across three cohorts [39]. Cohort 1 included patients who had not received recent platinum-based chemotherapy (*n* = 31). Cohorts 2 (*n* = 93) and 3 (*n* = 13) involved patients previously treated with platinum-based chemotherapy, with cohort 3 specifically focusing on those treated and asymptomatic BM. The ORR was 32% for Cohort 1, 21% for Cohort 2, and 23% for Cohort 3; the median PFS’ was 5.5 months, 3.7 months, and 4.3 months, and the OS was 14.4 months, 9.3 months, and 6.8 months, respectively. These results suggest that atezolizumab provides comparable ORR and OS benefits for patients with and without previously treated BM. Future research with a larger study cohort is warranted to validate the outcomes with pretreated BM (cohort 3).

## 5. Dual-Agent Immune Checkpoint Inhibitors in NSCLC with Brain Metastases

The process of combining CTLA-4 and PD-1/PD-L1 inhibitors, termed “dual-agent immunotherapy”, is emerging as an effective strategy for lung cancer with BM. This approach targets two immune checkpoints and may improve the immune response in the brain’s complex tumor biology. The immunosuppressive microenvironment in the brain has historically made BM less responsive to single-agent immunotherapy. Preclinical studies suggest that combined immunotherapy, by enhancing T-cell infiltration and reducing regulatory T-cells, could counter the resistance developed in ICI monotherapy due to alternative immune checkpoints [40,41,42]. However, there are limited data on the effectiveness of this approach in treating patients of NSCLC with BM.

### Nivolumab and Ipilimumab: Pivotal Clinical Trials and Survival Benefits

The nivolumab (anti-PD-1) and ipilimumab (anti-CTLA-4) combination has been extensively researched in various cancers and studied in NSCLC patients with BM. The following sections will provide an overview of the data from a single-agent ICI in lung cancer patients and research in melanoma patients with untreated BM.

The CheckMate 227 trial [28] conducted a post hoc analysis of 1166 chemotherapy-naïve NSCLC patients with a Tumor Mutational Burden (TMB) of ≥10 mt/Mb, including 135 patients with treated, asymptomatic BM (*n* = 69 randomized to ipilimumab plus nivolumab, *n* = 66 to platinum-doublet chemotherapy [pemetrexed, cisplatin, gemcitabine, and carboplatin]). Compared to chemotherapy alone, those who received combined immunotherapy and chemotherapy had a median OS of 18.8 months versus 13.7 months (HR 0.57). The ORR was 33% for the combination therapy group and 26% for the chemotherapy alone group. The duration of response (DoR) was also longer for the combination therapy group (24.9 months) compared to 8.4 months for those treated with chemotherapy alone. For patients without BM, the combination therapy group had a median OS of 17.1 months and a DoR of 19.6 months. In contrast, the chemotherapy alone group had a median OS of 13.9 months and a median DoR of 5.8 months. These findings demonstrate the activity of combination ICI therapy in patients with BM and suggest an added benefit compared to chemotherapy alone for this subgroup that mirrors the improvement seen in patients without BM.

The phase IIIb/IV CheckMate 817 trial was an exploratory analysis of 49 NSCLC patients with untreated BM treated with a fixed dose of nivolumab (240 mg every 2 weeks) and a weight-based dose of ipilimumab (1 mg/kg every 6 weeks) as a frontline therapy [29]. Those with untreated BM had a median OS of 12.8 months and a median PFS of 2.8 months. The ORR was 32.7%, and the median DoS was 12.8 months. This was a single-arm prospective trial where the intracranial benefits among patients with BM could not assessed, suggesting future prospective trials.

## 6. Combining Immune Checkpoint Inhibitors with Stereotactic Radiosurgery: A Synergistic Approach to Local Control and Survival

Radiation therapy, crucial in managing BM, has evolved to focus on improving local control while reducing neurocognitive side effects. Thus, there has been a shift from WBRT to SRS for treating solid tumors. SRS targets tumors and modulates the immune response by altering tumor cell antigen expression, stimulating interferon production, and inducing immunogenic cell death. This process creates an inflamed tumor microenvironment conducive to T-cell migration from the peripheral areas to the tumor site [43,44,45,46]. The rationale for combining SRS with ICIs lies in the synergistic enhancement of the immune response. Radiation is believed to act as a vaccine that releases neoantigen epitopes that sensitize the immune system. This effect is augmented by ICIs, which facilitate T-cell trafficking across the BBB, further weakened by SRS. This combination can lead to a systemic anti-tumor response, termed ‘abscopal effect’. While the optimal sequencing of SRS and ICIs is still under investigation, various retrospective studies, particularly in NSCLC, have shown promising outcomes (Table 2). Patients with melanoma BM receiving both treatments have been reported to have longer survival compared to those with immunotherapy alone, depending on the sequence of therapies, highlighting the need to evaluate these combinations closely. For example, SRS may be given after immunotherapy to prevent BM. However, SRS before ICI may be more valuable considering its effective treatment of symptoms and relief of the need for steroids, which should be avoided early when using ICI [47]. However, well-designed randomized trials with the appropriate stratification for factors influencing outcomes other than SRS and ICI are needed to optimize therapy in lung and other cancers that frequently metastasize to the brain.

Studies in NSCLC suggest that ICI with concurrent SRS improve OS compared to sequential treatment [48,49,50]. Schapira and colleagues retrospectively analyzed the effects of timing when administering SRS and anti-PD-1 immunotherapy (simultaneous treatment, before or after immunotherapy) in 37 NSCLC patients with BM [51]. Significant improvements in one-year OS for patients treated with ICI and SRS together (87.3%) were seen compared to those treated with SRS before (70.0%) or after ICI (0%). Concurrent treatment was also associated with a lower incidence of distant brain failure (new BM or tumor progression beyond the previously irradiated area in the brain) compared to sequential treatment. Enright et al. evaluated 77 NSCLC patients with BM, comparing 33 patients receiving both SRT and ICI with 44 patients receiving only SRT [52]. Findings revealed that combination therapy resulted in lower distant brain failure, fewer neurological deaths, and improved OS, with a median OS of 13.9 months across the cohort. Additionally, the ARIO study compared SRT with ICI (*n* = 100) or without ICI (*n* = 50) in NSCLC patients with BM [53]. Combination therapy significantly improved intracranial local progression-free survival (iLPFS), with patients receiving combination therapy showing slightly higher iLPFS rates at 89.5% versus controls (83.9%). However, there were no significant differences in OS rates at 6 (79.4% vs. 64.5%) and 12 months (79.4% vs. 64.5%) after SRT between the groups. Similarly, Kotecha et al. found that combining SRS with immunotherapy led to higher intracranial response rates (73%) and prolonged OS, especially with lower or no steroid use (25.1 months without dexamethasone, 10.2 months with ≤60 mg, and 10.2 months > 60 mg) [54]. Thus, combining radiotherapy with ICI may be a viable approach underpinned by strong preclinical evidence, yet further prospective research is needed to strengthen these claims.

**Table 2:** Clinical Outcomes of ICI and Radiotherapy Combinations in NSCLC Brain Metastases.

Trial Name	Study Design	Patient Population (with BM)	Intervention Arm	Control Arm	PD-L1	iORR (num. %)	mPFS	mOS
Studies with ICI ± Radiotherapy Combinations								
Ahmed KA, et al. [50]	Retrospective	17	RT (SRS or FSRT) ± anti-PD1/PD-L1	None	NR	NR	NR	17.9
Schapira E, et al. [51]	Retrospective	37	SRS + anti-PD1/PD-L1 (concurrent and sequential)	None	Any	NR	NR	17.6
Eright TL, et al. [52]	Retrospective	33 (SRT + ICI), 44 (SRT)	SRT ± anti-PD1/PD-L1 inhibitors (A)	SRT (B)	A: median PD-L1 10%, B: median PD-L1 30%	NR	NR	13.9
Soccianti S, et al. [53]	Retrospective	100 (RT + ICI), 50 (RT)	RT (SRS, SRT or HFSRT) ± ICI (concurrent and sequential)	SRT	Any/None	NR	NR	1-Year OS: SRT + ICI (64.5%) and SRT (67.5%)
Chen et al. [55]	Retrospective	157	Concurrent SRS + ICI	None	Any	NR	1-year intracranial PFS rate: 88%	NR

A and B: Denote different arms within the study (e.g., treatment or control arms as defined in the study protocol; NR: Not Reported.

## 7. Immune Checkpoint Inhibitors and Chemotherapy: A Combined Strategy for Treating NSCLC with Brain Metastases

ICIs combined with chemotherapy are the standard first-line therapy for metastatic NSCLC patients. This approach, supported by data from multiple large, randomized phase II and III clinical trials, marks a significant advancement in lung cancer therapy as it leverages the immunomodulatory effects of chemotherapeutic drugs along with the targeted action of ICIs (Table 3). To date, this combination has generated the most robust anti-tumor immune response in lung cancer patients [56,57].

A network meta-analysis of 12 phase III trials with 9236 patients with metastatic NSCLC examined the effectiveness of frontline therapies incorporating at least one ICI, with or without chemotherapy [57]. Combining chemotherapy with pembrolizumab or atezolizumab was effective compared to chemotherapy alone, ICI combination, or ICI monotherapy, suggesting that chemotherapy enhances the effectiveness of ICIs as the primary treatment for advanced NSCLC. Moreover, the efficacy of combination therapy extends across diverse patient groups, showing promising results for squamous and non-squamous NSCLC patients and across all PD-L1 expression levels, including high, negative, and intermediate PD-L1 (1% ≤ PD-L1 < 50%) groups [57].

### 7.1. Pembrolizumab Plus Chemotherapy: Pivotal Clinical Trials and Survival Benefits

The KEYNOTE-189 trial, alongside a pooled analysis by Powell et al. of three phase III KEYNOTE clinical trials, offers comprehensive insights into the efficacy of pembrolizumab combined with chemotherapy in NSCLC patients, including those with BM [59,64]. The phase III KEYNOTE-189 trial evaluated 616 previously untreated non-squamous NSCLC patients, including 108 patients with asymptomatic BM and any level of PD-L1 expression [59]. The trial compared pembrolizumab vs. placebo with platinum-based chemotherapy and pemetrexed. Patients with BM in the pembrolizumab group (*n* = 73) had a median PFS of 6.9 months and a median OS of 19.2 months, significantly better than the 4.7 months (mPFS) and 7.5 months (mOS) observed in the placebo group (*n* = 35).

Results from the pooled exploratory analysis by Powell et al. further validate these findings [64]. The analysis involved 171 patients with baseline BM out of 1298 from the three KEYNOTE trials. The KEYNOTE-189 and -407 studies enrolled patients with untreated, asymptomatic BM below 1.5 cm that did not require corticosteroids. Combined pembrolizumab and chemotherapy substantially improved the outcomes compared to chemotherapy alone in patients with and without BM. Notably, in those with BM, the combined treatment significantly extended the median OS to 18.8 months compared to just 7.6 months with chemotherapy alone. The median PFS was also prolonged to 6.9 months with combination therapy, compared to 4.1 months with chemotherapy alone [64]. The analysis was exploratory and did not adjust for multiple comparisons, limiting these findings. Most patients had asymptomatic, small BM, and a small percentage had previously used brain radiotherapy, limiting the applicability to the general NSCLC patient population. The evaluation of intracranial responses was not feasible in the analysis, as brain lesions were classified as non-target lesions. These findings demonstrate the benefit of pembrolizumab combined with chemotherapy in improving survival outcomes for NSCLC patients with and without BM.

### 7.2. Nivolumab and Ipilimumab Plus Chemotherapy: Pivotal Clinical Trials and Survival Benefits

The CheckMate 9LA study was a phase III trial evaluating a novel regimen in 1150 treatment-naïve NSCLC patients without known sensitizing EGFR/ALK genomic tumor aberration. The trial compared the effectiveness of nivolumab and ipilimumab with chemotherapy alone, including patients with treated, asymptomatic CNS metastases and any level of PD-L1 expression [61]. There was a significantly longer median OS for the immunotherapy group (19.9 months) than chemotherapy (7.9 months). Further, an ORR of 38.2% for the immunotherapy-based treatment was found compared to 24.9% for the chemotherapy arm. This combination is now FDA-approved for treating metastatic or recurrent NSCLC as a first-line treatment, excluding those with EGFR or ALK genomic tumor aberrations.

### 7.3. Atezolizumab Plus Chemotherapy: Pivotal Clinical Trials and Survival Benefits

The ATEZO-BRAIN study was a phase II trial investigating atezolizumab with carboplatin and pemetrexed in 40 non-squamous NSCLC patients with stable untreated BM either without neurological symptoms or managed with anticonvulsants or a maximum of 4 mg daily of dexamethasone, without considering PD-L1 expression [58]. Up to 55% of the patients were on corticosteroids at the start of the study. After a median follow-up of 31 months, an intracranial response rate of 40% was reported based on the Response Assessment in Neuro-Oncology Brain Metastases (RANO-BM) and a systemic response rate of 47.5% based on the Response Evaluation Criteria in Solid Tumors (RECIST v1.1). The median systemic PFS was 8.9 months, the intracranial PFS was 6.9 months, and the median OS was 13.6 months. There were no differences in the response rates of OS based on PD-L1 expression or corticosteroid use. While the 2-year OS rate was observed to be higher in patients with PD-L1 expression ≥1% (40.0%) compared to those with lower levels (16.7%), these differences were non-significant, highlighting the need for cautious interpretation. These findings suggest that while there may be trends worth further investigation, the current data do not conclusively demonstrate that this treatment approach should be limited to patients with higher PD-L1 expression.

## 8. Role of Steroids in ICI-Based Therapies in Brain Metastases

The interplay between steroids and ICIs in BM patients is crucial, given the routine use of steroids in these cases and their contrasting effects on the immune system. Studies indicate that steroids may reduce the survival benefits of ICIs, with 40% of ICI trials excluding steroid-treated patients. Studies have shown that steroids are associated with poorer OS and PFS in BM patients receiving ICIs [65]. A meta-analysis examining metastatic cancer patients, predominantly those with melanoma and NSCLC treated with ICIs, showed that steroid usage correlated with significantly worse OS (HR = 1.54) and PFS (HR = 1.34) [66].

Arbour and colleagues conducted a study investigating the effect of baseline steroid therapy on the effectiveness of ICIs [67]. The study included 640 patients with advanced NSCLC who were treated with PD-L1 inhibitors, 90 of whom were taking a daily dose of ≥10 mg prednisone when they began ICI therapy. Patients who received both prednisone and ICIs had a worse prognosis than those who did not. Other studies echoed these findings [68,69], suggesting that early steroid use was associated with poorer outcomes in NSCLC BM patients treated with ICIs. The observed diminished effectiveness of PD-(L)1 inhibitors in patients on steroids might be due to the immunosuppressive nature of steroids counteracting the mechanism of action of ICIs. Additionally, the poor prognosis of patients at baseline (due to larger lesions) requiring steroids could also contribute to this outcome. Research by Kotecha et al. reports that in patients with low or no steroid use, combining SRS with ICI led to higher ORR (73%) and prolonged OS (25.1 months vs. 10.2 months with ≤60 mg dexamethasone), indicating a more favorable scenario when steroids are minimized [54]. Further prospective studies are warranted to clarify the impact of corticosteroids on ICI effectiveness in NSCLC BM.

## 9. Combining Immune Checkpoint Inhibitors and VEGF Inhibitors: A New Frontier in NSCLC Brain Metastasis Management

In advanced NSCLC, combining ICIs with anti-VEGF agents represents a notable step forward in cancer therapy. Anti-VEGF agents modulate the tumor microenvironment by impeding angiogenesis, which is a critical process for tumor proliferation and metastatic spread. They also potentiate immune-mediated tumor suppression by upregulating mature dendritic cells and augmenting antigen presentation, activating T lymphocytes [70]. Conversely, VEGF plays a pivotal role in angiogenesis and contributes to immunosuppression within the tumor microenvironment by impairing T-cell differentiation and fostering an environment conducive to tumor evasion [70]. By inhibiting immune checkpoints like PD-1 and CTLA-4, ICIs can boost the body’s natural anti-tumor activity. Additionally, ICIs contribute to normalizing tumor vasculature, further aiding immune cell efficacy.

Clinical studies have shown that combined ICI and anti-VEGF treatment improves PFS and OS compared to monotherapies. However, challenges in patient selection and drug resistance persist. Bevacizumab, an anti-angiogenic agent approved for metastatic non-squamous NSCLC, complements atezolizumab’s function by counteracting VEGF-induced immunosuppression. Results from the IMpower150 trial highlight the efficacy of the atezolizumab, bevacizumab, carboplatin, and paclitaxel (ABCP) combination in reducing the development of new BM in patients with NSCLC [62]. After a 32.4-month follow-up, the ABCP regimen was associated with a lower incidence of new BM formation (7.0%) compared to atezolizumab with carboplatin and paclitaxel (ACP, 11.9%) and bevacizumab with carboplatin and paclitaxel (BCP, 6.0%). Findings from this study provide valuable insights into the potential role of bevacizumab in delaying or preventing the progression of BM in NSCLC.

## 10. Conclusions

This review emphasizes the critical need for a personalized, multidisciplinary treatment strategy for BM from NSCLC. Despite advancements, there remains no universally accepted guidelines for the sequence or combination of treatments, especially concerning the integration of ICIs with traditional therapies. Currently, the standard treatment approach for BM involves a combination of neurosurgical intervention, radiation therapy, and systemic therapies like chemotherapy, targeted therapy, and immunotherapy. ICIs, especially those targeting PD-1/PD-L1, have notably improved outcomes in NSCLC with BM, as demonstrated by increased OS and PFS in various clinical trials. The field is expanding to include newer checkpoints, such as lymphocyte activation gene 3 (LAG-3).

In addition to ICIs, innovative therapies like bispecific antibodies, which target two different tumor antigens to enhance the immune response, and other novel modalities such as cancer vaccines, CAR-T cell therapy, and oncolytic virus therapy are being studied for their efficacy in various cancers, including NSCLC and BM. These novel approaches offer potential breakthroughs, but their application in BM requires further investigation to evaluate long-term safety and efficacy.

While combining ICIs with radiotherapy, chemotherapy, and anti-VEGF agents, such as bevacizumab, holds promise in augmenting treatment efficacy, these strategies must be balanced with the risks of neurotoxicity, cognitive side effects, and radiation necrosis. Neurological symptom management with steroids, neurosurgery, or SRS, while considering the potential impact of these interventions on the effectiveness of eventual ICIs, is crucial. This highlights the ongoing challenge of steroid use, which may impair ICI efficacy and calls for strategies that minimize reliance on corticosteroids. Optimal methods of transitioning steroid-dependent patients to steroid independence are critical.

Although clinical trials have a limited representation of BM patients, further research is necessary based on the findings. This includes prospective clinical trials involving BM patients, providing more robust data for treatment strategies. Additionally, real-world data—augmented with artificial intelligence and advanced analytics—can identify critical gaps in patient management, offering insights into optimizing clinical decision-making. Future clinical trials should further explore combination therapies, including ICIs with chemotherapy and anti-VEGF agents. Research into the sequencing and timing of these treatments, particularly radiotherapy and immunotherapy, remains a priority to realize the potential of combination strategies fully.

## Figures and Tables

**Table 3:** Pivotal Trials of ICI–Chemotherapy and ICI–VEGF Inhibitor Combinations in NSCLC Brain Metastases.

Trial Name	Study Design	Patient Population (with BM)	Intervention Arm	Control Arm	PD-L1	iORR (num. %)	mPFS	mOS
Studies with ICI–Chemotherapy Combinations								
ATEZOBRAIN Trial [58]	Phase II	40 (untreated BM)	Atezolizumab, carboplatin and pemetrexed	None	Any	47.5	8.9	13.6
KEYNOTE-189 [59,60]	Phase III	73 (A), 35 (B) (asymptomatic BM)	Pembrolizumab, platinum and pemetrexed (A)	Platinum and pemetrexed (B)	Any	47.6 (A), 18.9 (B)	6.9 (A), 4.7 (B)	19.2 (A), 7.5 (B)
CheckMate 9LA [61]	Phase III	64 (A), 58 (B) (treated asymptomatic CNS BM)	Nivolumab, ipilimumab, and chemotherapy (A)	Chemotherapy (B)	Any	38 (A), 25.4 (B)	NR	19.9 (A), 7.9 (B)
**Studies with ICI–VEGF Inhibitors Combinations**								
IMpower150 trial [62]	Phase III (exploratory analysis)	1202 (ITT population)	Atezolizumab, bevacizumab, carboplatin, and paclitaxel (A); Atezolizumab with carboplatin and paclitaxel (B)	Bevacizumab, carboplatin, and paclitaxel (C)	Any	NR	NR	NR
Sugawara et al. [63]	Phase III	550	Nivolumab, bevacizumab and chemotherapy (carboplatin and paclitaxel) (A)	Placebo bevacizumab and chemotherapy (carboplatin and paclitaxel) (B)	Any	61.5% (A), 50.5 (B)	12.1 (A), 8.1 (B)	25.4 (A), 24.7 (B)

A–C: Denote different arms within the study (e.g., treatment or control arms as defined in the study protocol; NR: Not Reported.
